# Understanding the Lives of Aboriginal and Torres Strait Islander Women with Traumatic Brain Injury from Family Violence in Australia: A Qualitative Study Protocol

**DOI:** 10.3390/ijerph20021607

**Published:** 2023-01-16

**Authors:** Michelle S. Fitts, Jennifer Cullen, Gail Kingston, Yasmin Johnson, Elaine Wills, Karen Soldatic

**Affiliations:** 1 Institute for Culture and Society, Western Sydney University, Parramatta, NSW 2751, Australia; 2 Menzies School of Health Research, Charles Darwin University, Alice Springs, NT 0871, Australia; 3 Australian Institute of Tropical Health and Medicine, James Cook University, Cairns, QLD 4878, Australia; 4 Synapse Australia, Brisbane, QLD 3356, Australia; 5 College of Healthcare Sciences, James Cook University, Cairns, QLD 4878, Australia; 6 Townsville Hospital and Health Service, Townsville, QLD 4814, Australia; 7 School of Social Sciences, Western Sydney University, Parramatta, NSW 2751, Australia

**Keywords:** women, traumatic brain injury, violence, Australia, Aboriginal and Torres Strait Islander, care systems

## Abstract

Globally, there is growing recognition of the connection between violence and head injuries. At present, little qualitative research exists around how surviving this experience impacts everyday life for women, particularly Aboriginal and Torres Strait Islander women. This project aims to explore the nature and context of these women’s lives including living with the injury and to identify their needs and priorities during recovery. This 3-year exploratory project is being conducted across three Australian jurisdictions (Queensland, Northern Territory, and New South Wales). Qualitative interviews and discussion groups will be conducted with four key groups: Aboriginal and Torres Strait Islander women (aged 18+) who have acquired a head injury through family violence; their family members and/or carers; and hospital staff as well as government and non-government service providers who work with women who have experienced family violence. Nominated staff within community-based service providers will support the promotion of the project to women who have acquired a head injury through family violence. Hospital staff and service providers will be recruited using purposive and snowball sampling. Transcripts and fieldnotes will be analysed using narrative and descriptive phenomenological approaches. Reflection and research knowledge exchange and translation will be undertaken through service provider workshops.

## 1. Introduction

Exposure to violence has serious health outcomes for women [1], with the elimination of violence against women and children a recognised national priority in Australia [2,3]. Due to the recurrent nature of family violence [4], women are vulnerable to sustaining injuries that impact upon the functioning of their brain. Traumatic brain injury (TBI) is defined as damage to, or alteration of, brain function due to a blow or force to the head [5]. A subset of acquired brain injury (ABI), the experience of TBI is unique and can consist of various short- and long-term cognitive impacts as well as psychological and physical consequences. These changes can include memory loss, difficulty with motivation, lack of insight, sensory and perceptual problems, posttraumatic epilepsy, fatigue and sleep difficulties, mood changes, and anxiety [6,7,8,9]. Even mild TBI is a risk factor for the development of early onset dementia and other chronic health conditions [10]. Although there has been growing recognition of the intersection between family violence and head injury both in Australia and worldwide [11,12], this has yet to translate into significant research action to listen to the voices of Aboriginal and Torres Strait Islander women who have acquired a head injury or been diagnosed with a TBI connected to family violence. The development of new knowledge is critical for informing robust, evidence-based family violence, disability and health policy, and practice.

Indigenous women in Australia and other settler nations such as Aotearoa New Zealand and the United States of America experience higher levels of violence than their non-Indigenous counterparts. Compared to other Australian women, Aboriginal and Torres Strait Islander women are more likely to be hospitalised due to violence and die due to injuries sustained from violence [13,14,15]. Concerningly, Aboriginal and Torres Strait Islander women in remote and very remote areas are more likely to be hospitalised for family violence than their urban counterparts (26.5 versus 2.8 per 1000) [13]. Violence against Aboriginal and Torres Strait Islander women is perpetuated by men of all cultural backgrounds including non-Indigenous men. In response to these high rates of violence, Aboriginal and Torres Strait Islander community leaders have led actions to prevent and address family violence [16,17]. Such trends are similarly reflected in assault-related head injury presentations [5,18]. One study found Aboriginal and Torres Strait Islander women are hospitalised with a head injury due to assault (1999–2005) 69 times the rate of other Australian women [5]. Factors such as alcohol are associated with TBI experienced by Indigenous peoples [19]. However, further research to understand the potential contribution of alcohol with TBI occurrence is warranted [20]. Incomplete and inaccurate data collection, together with the underreporting of violence by survivors of violence, also suggests that the published rates are, at best, an indication of the extent of violence experienced by women [21,22,23].

The terms domestic violence, family violence, and intimate partner violence are often used to describe violence against women. Although these terms are used interchangeably, the preferred term for Aboriginal and Torres Strait Islander communities tends to be family violence, as it encapsulates the extended nature of Aboriginal and Torres Strait Islander families and the kinship relationship within which a range of forms of violence occur [24]. For Aboriginal and Torres Strait Islander communities, family violence is a complex issue and must be seen in the context of wide-scale colonisation involving oppression, dispossession, massacres, the removal of children, and the loss of linguistic and cultural authority combined with the direct consequences of these policies on Aboriginal and Torres Strait Islander communities. Impacts include intergenerational trauma, economic and housing stress, unemployment as well as alcohol and other drug misuse [25,26,27].

Despite Aboriginal and Torres Strait Islander women experiencing high rates of head injury connected to family violence [5], research to document and examine their lived experiences remains scarce. Two reviews of TBI research with Indigenous populations identified a lack a studies investigating the lived experiences of Indigenous women in Aotearoa New Zealand, Australia, Canada, and the United States of America with head injury connected to violence [19,28]. Since then, two large scale Australian studies, ‘The transition from hospital to home’ (2016–2018; Queensland and Northern Territory) and ‘Healing Right Way’ (2017–2021; Western Australia), focused only on the transition period of Aboriginal and Torres Strait Islander peoples, which consists of hospital admission through to discharge and return to community and country [29,30,31]. Culturally responsive community rehabilitation models and resources were developed and implemented from these works [32,33]. Despite these promising advancements, the research area has narrowly focused on those patients who accessed hospital care alone. Thus, the models of rehabilitation developed are general in nature and may not recognise the unique issues that emerge when TBI is a direct outcome of family violence. Women who live with an acquired head injury as a result of family violence may not access health care, with fear shaping their daily lives and ability to seek help and access resources [34]. Other barriers to accessing health care include marginalisation, shame and stigma, and worries about confidentiality in tight-knit communities [31,35,36].

### Study Aims 

Although there are distinct bodies of literature examining family violence and TBI that demonstrate Aboriginal and Torres Strait Islander women experience high rates of both phenomena, the intersectionality of TBI and family violence has been overlooked [34]. Appropriate, effective, and equitable access to service providers that support and address the unmet needs and priorities of these women and their families has the potential to reduce the high incidence of head injury rates experienced by women and improve their health and well-being across their life course. With the increased recognition of both family violence and TBI in national initiatives [2], it is now critical to document and understand the beliefs, perceptions, and experiences of Aboriginal and Torres Strait Islander women with acquired head injury in the context of family violence. The aims of this project are to:

- Document the nature and context of the lives of Aboriginal and Torres Strait Islander women who are living with the consequences of a head injury caused through family violence.
- Map and compare populations of Aboriginal and Torres Strait Islander women living with a head injury and key issues for these populations across the three contrasting locations.
- Enrich the theoretical and applied understandings of the growing TBI population of Aboriginal and Torres Strait women and their experiences of change and reconstruction of self-identity after a head injury acquired through family violence.
- Develop outputs (such as resources) and a rigorous theoretical framework to inform government (national, state, local level) policies and programs.

## 2. Methods

### 2.1. Setting

This project will be undertaken at three sites in three Australian jurisdictions (Queensland, Northern Territory, and New South Wales). Family violence research demonstrates recognition of the heterogeneity in the experiences of survivors and service providers across regional and remote areas [37]. Therefore, the involvement of the three locations will help to better understand the effects of social and geographical isolation on the ability of women to disclose, report, and seek help about family violence. In addition, these sites were selected to understand the impact of different programs, policies, and geography for women who live with head injury and the services who support them. Together, this will allow for a comparative analysis and close examination of three different jurisdictions. The population of project location 1 is approximately 25,000, with an Aboriginal and Torres Strait population of 4361 (17.6%). The population of project location 2 is approximately 230,000 with 18,008 (7.9%) of residents identifying as Aboriginal and/or Torres Islander [38]. In project location 3, approximately 1.4% (*n* = 13,426) of the total population (*n* = 936,433) identify as Aboriginal and/or Torres Strait Islander.

### 2.2. Conceptual and Theoretical Innovation

This project will draw upon socially-embedded phenomenology to develop a richer and more sophisticated understanding of the way acquired head injury disrupts a person’s embodied being in the world. Exploration of the social creation of impairment through inequality, deconstructing the cultural construction of impairment, and analysing the personal significance of impairment identities is required to build upon existing research knowledge [39]. While previous TBI studies have made important contributions to understanding disability through TBI, under the medical model, there is a strong focus on outcomes that are valued within a Western framework. Indeed, under the medical model, the emphasis is on ‘fixing’ the impairment, so that people can ‘function’ in society as active participants (such as returning to employment) [40]. A general assumption is made that illnesses and disabilities are universal and invariant to the cultural and social contexts in which they exist [41]. In turn, the research and guidance for workforces who support these women are limited, with the intersections of cultural identity potentially complicating what is already a complex issue. Within this project, an understanding of how different intersectionalities [42] such as gender, cultural belonging, and geographical location contribute to the women’s experiences of services and systems after acquiring a head injury through family violence will be explored.

### 2.3. Research Governance 

Aligning with the national research guidelines [43], the project will ensure Aboriginal and Torres Strait Islander peoples are involved in all aspects of the project. An advisory group consisting of members that represent a selection of service providers, community groups and hospitals participating in the project as well as representatives from national disability advocacy groups will meet twice a year (by teleconference). A critically reflective process will be completed at each site, entailing ongoing adjustments to the research process and incorporating iterative feedback into the research approach undertaken at each site [44,45]. This will enable the process to be locally guided to ensure that processes are responsive to local community requirements and local cultural protocols across the project, and simultaneously draw out comparative and contrasting aspects across each of the sites to inform a comprehensive interpretative analysis of the findings [46]. Individual advisors including Aboriginal and Torres Strait Islander women with lived experience of violence-related TBI will also help guide the project. 

### 2.4. Definition of Traumatic Brain Injury 

There is not one consistent definition used to define a TBI [47]. The inclusion criteria for the project are broad to include women with different severity levels of TBI. The project will define mild TBI (which can also be referred to as a head injury) as trauma to the head that is severe enough to cause neurological symptoms (including sensitivity to light, headache, and nausea). A moderate to severe TBI can be identified by one of the following: (1) loss of conscious for any duration, OR (2) post-traumatic amnesia > 24 h, OR (3) injury verified on a computerized tomography (CT) scan or magnetic resonance imaging (MRI). All major hospitals in the three project locations have CT and MRI facilities. The broad criteria also account for the suite of factors that can reduce accessibility to health care and specialist services following a TBI for women living in regional and remote communities [48,49].

### 2.5. Participants and Recruitment 

The participants are outlined in Table 1. Aligning with the qualitative nature of the project, a non-probability sampling approach will be adopted, and no sample size calculation will be carried out [50]. The sampling aims to include information-rich cases and achieve in-depth understanding of the phenomena being explored rather than striving to meet a specific (statistically determined) sample size [51]. Sampling aims to reach a diverse range of participants (including age and location) and achieve data saturation across themes. The sample size of each group has also been determined by practical considerations including timeframes of qualitative fieldwork and reasonable workload requests for service providers who will support the recruitment of women who have experienced a head injury through family violence.

#### 2.5.1. Women Who Have Experienced a Head Injury through Family Violence 

To preserve the safety of women, the research team will work closely with frontline service providers and community groups within each project site to purposefully sample women who fit the criteria. The broad inclusion criteria for this study are women who: (1) identify as Aboriginal and Torres Strait Islander; (2) are aged 18+; and (3) have experienced a head injury (or been diagnosed with a TBI) as a direct consequence of family violence. The recruitment of women will continue until data saturation is reached [52]. Frontline service providers are well-equipped to identify potential participants, with some service providers (such as legal and health services) having access to medical discharge summaries that confirm whether their patient or client would meet the study criteria. Other services have active risk management plans and communication strategies in place with their clients, which makes them well-equipped to have knowledge and awareness of the different factors that may place each client at greater or lesser risk to participate in the study. Nominated service staff (such as women’s groups coordinators, medical practitioners, case workers, and lawyers) will identify patients or clients who meet the eligibility criteria. Once a clinician has identified a patient that meets the eligibility criteria, a member of the research team will be notified. To ensure consent is conducted in an appropriate manner, the research team member will approach the patient or client with the assistance of an Aboriginal Interpreter Translator (where necessary) to explain the study fully. Potential participants will be provided with written information about the study, a short video about the project, face-to-face discussion with a research team member, and given an opportunity to ask questions about the project. To support nominated staff from service providers to identify women who meet the criteria, TBI education sessions will be delivered to participating service providers by two Aboriginal and Torres Strait Islander educators from a national brain injury organisation [53]. Aboriginal Interpreter Translators will also be employed to assist with data collection. 

#### 2.5.2. Family Members and Carers

Women who have experienced a head injury through family violence will be asked to nominate a family member or carer (aged 18+) to take part in an interview. Family members and carers can nominate to take part in an interview or small discussion group. Women with acquired head injury related to family violence can participate in the study without nominating a family member or carer. 

#### 2.5.3. Hospital Staff

Direct invitations will be made to hospital staff and a ‘snowballing’ approach will also be used for recruitment, where participants will be able to recommend other staff to approach. A variety of disciplines will be targeted with the aim to include individuals who have lived experience of working with women who present to the hospital with head injury connected to family violence, their families, and perpetrators of family violence, and those who provide medical treatment to women who have sustained a head injury. Potential staff that will participate in an interview for the project include Indigenous hospital liaison officers, Aboriginal health workers, specialists, nurses, occupational therapists, physiotherapists and social workers.

#### 2.5.4. Service Professionals, Community Groups, and National Advocacy Groups

Frontline workers, community-based groups, and advocacy groups will be invited to participate in an individual interview or small discussion group. Frontline workers will represent primary health care (both Aboriginal community-controlled and government), the legal and justice sector (including magistrates, lawyers, victims support), housing and crisis accommodation, disability support services, and family violence services. Examples of community groups include women’s and Elder groups. Representatives from national and regional disability and brain injury advocacy groups will also be invited to participate in an individual interview. This previously used approach [54] involves the selection of experts based on their knowledge and experience of the core research issue [55]. Prioritising contact with Aboriginal and Torres Strait Islander community groups and organisations, a list of potential participants will be developed by the research team. Sampling will occur through the networks of the research team and a ‘snowballing’ approach, with participants asked to recommend other relevant individuals and agencies to participate in the research [51]. As presented in Table 2, the interview protocol for service professionals aims to understand the delivery and practice of service models as well as to understand the knowledge of service professionals related to TBI and family violence.

### 2.6. Data Collection

Interviews and discussion groups will be conducted by Aboriginal and non-Indigenous research team members. A semi-structured interview guide covering the topics listed in Table 2 will be used, but questions will also be informed by observations and new topics raised by the participant. Participants will have the opportunity to ‘tell their story’. These methods have been rigorously applied in previous research with Aboriginal and Torres Strait Islander women and are drawn upon here as they can: (a) capture grounded subjective experiences and practices occurring locally and (b) effectively support the participation of highly marginalised groups who have a diverse range of skills, knowledge, and educational attainment [56]. Yarning is also a feature of Aboriginal and Torres Strait Islander convention for passing on information through informal conversations, reflecting the oral traditions that support the transmission of knowledge among Aboriginal and Torres Strait Islander peoples [57]. The interview guide was developed through a multi-phase process involving - aligning the interview questions with research questions, receiving feedback on the interview schedules, and piloting of the interview schedules [58].

### 2.7. Workshops

Once data have been collected from across all studies, service providers will be invited to participate in one-day workshops. Based on participatory models of qualitative research methods, discussion questions will be designed to elicit in-depth information about the existing knowledge and gather information and recommendations for the next steps in research, practice, and knowledge dissemination. Breakout groups will include at least one representative from different types of service providers and community groups to achieve triangulation of the data. Session summaries of the workshops with services will be presented to the collective group at each location to identify key themes and prioritise next steps. Discussions will be recorded in written and audio formats.

### 2.8. Data Analysis

All audio recorded interviews, discussion groups, and workshops will be transcribed verbatim. Transcripts, fieldnotes, and observations will be managed with NVivo 12 [59]. The transcripts will be analysed using a combination of narrative and descriptive phenomenological analyses. The aim of descriptive phenomenology is to describe particular phenomena, or the appearance of things, as lived experience [60]. The process is inductive and descriptive and seeks to record experiences from the viewpoint of the individual who had them without imposing a specific theoretical or conceptual framework on the study prior to collecting data [61]. The narrative analysis will focus on sense-making and Aboriginal and Torres Strait Islander women’s changing identity and role post TBI from family violence. The two methodological approaches complement each other in terms of gaining knowledge of ‘breadth’ (narrative identity) and ‘depth’ (lived experiences), giving some support for a philosophical position that shows a person as both an active and passive agent, constructively making sense of their narrative identity as well as being constructed by their lived experiences. A constant comparative technique will be employed to systematically organise, compare, and understand the similarities and differences across the different participating groups and field sites, critically enriching the analysis and providing a substantive basis for theoretical extrapolation and affording critical points of comparative analysis across each of the locations.

### 2.9. Ethics

Ethics approval for this research has been obtained from the Central Australian Human Research Ethics Committee (CA-21-4160), Western Sydney University Human Research Ethics Committee (H14646), Townsville Hospital and Health Service Human Research Ethics Committee (HREC/QTHS/85271 and HREC/QTHS/88044), and the Aboriginal Health and Medical Research Council of New South Wales Human Research Ethics Committee (1922/22).

### 2.10. Consent 

Participants will provide voluntary written informed consent. For women who experience severe impairment or are under a guardianship order, consent from a proxy for research participation from a person responsible for the person (such as a carer or guardian) will be sought. Participants may withdraw from the study at any time before dissemination of the findings, except for the discussion group participants, as it will be difficult to identify individual voices in the recording and transcript. Discussion group participants will be informed of this before consenting to the study. Participants will not be deceived in any way about the study objectives. All information regarding the study will be provided verbally and in writing prior to the interview. To minimise the risk of stigmatisation of potential participants and to ensure information regarding the study is understood fully by the participants, a flipchart that uses images and plain, easy English to describe key aspects of the project (e.g., what is a brain injury, reasons for the study, and participant rights) as well as video resources developed in a previous TBI study will be used [33].

### 2.11. Potential Benefits and Risks

Self-awareness plays an essential role in TBI rehabilitation and can impact motivation, safety, and rehabilitation goals during recovery [62]. Through self-exploration of their lived experiences, some participants may be able to fully explore their experiences, more fully explore their circumstances, and may also gain a new perspective. However, the recall of traumatic experiences by women and their family members may also cause discomfort or distress. Drawing upon the guidelines for working with women who have experienced family violence, all decisions within the research process will be driven by an awareness of the safety and ethical considerations [63,64,65]. Several strategies will be implemented to minimise any potential risk, and to identify the different levels of risk, of the women being identified as participating in the study. Promotion and recruitment through only service providers recognises the importance of recruiting women that are already connected with one or more of the participating services with access to ongoing support. Service providers have an existing awareness of the current life circumstances of the women referred to the project (e.g., if women are living in a high-risk environment). Existing connections between services and women who have experienced family members will also enable the research team to complete immediate referrals (with permission of the participant) back to the service for support should the women disclose that they are at risk of violence or have been identified by other community members as participating in the study. Other safeguards implemented for the safety and well-being of women and carers/support persons who take part in the study include the organisation of an experienced counsellor when interviews and discussion groups are conducted, and follow-up contact with each participant shortly after data collection. 

### 2.12. Dissemination

A dissemination plan implemented within the project will ensure that the research findings and dissemination activities are controlled by Aboriginal and Torres Strait Islander peoples and service providers and ensure that the findings are disseminated throughout the course of the project in appropriate formats for the stakeholders. Stakeholders include research participants, government and non-government service providers, the health and legal sectors, state/territory and federal policymakers, the academic community, and advocacy groups. Findings will be disseminated through conference presentations and peer-review publications. Further dissemination activities will be determined in partnership with advisory groups and other service providers through the recommendations made in the workshops. Some of the expected dissemination formats include:

- Research translation workshops with services and hospital representatives as well as community presentations. Project partner policy papers for use by advocates involved in the project.
- Incorporating art, visual media, and other media to present essential information for community members about the key findings.
- Translating all findings into easy English versions of the final report.

While this study will directly inform policy and practice within Queensland, the Northern Territory, and New South Wales, the findings will be disseminated to other relevant states/territory service providers, government ministers, and advocacy groups beyond these jurisdictions to ensure that an applicable national-level strategy can be shared. Publications will adhere to the CONSolIDated critERia for strengthening the reporting of health research involving Indigenous Peoples (the CONSIDER statement) [66] as well as the Consolidated Criteria for Reporting Qualitative Research [67].

## 3. Conclusions

This qualitative project will comprehensively explore and document the strengths, challenges, and nuances in the day to day lives of Aboriginal and Torres Strait Islander women with an acquired head injury connected to family violence. Through partnerships with key services, the evidence generated will enable service providers that work with these women to better develop and tailor their services, programs, and workforce to support Aboriginal and Torres Strait Islander women and their families. The evidence may also help to inform resource allocation and provide vital information for governments to support the planning and development of equitable, holistic, appropriate care, and support that reflects the needs and priorities of Aboriginal and Torres Strait Islander Australian women experiencing head injury in the context of family violence. The evidence generated from this project is a critical step in addressing the unacceptable rates of head injury as a result of family violence among Aboriginal and Torres Strait Islander women. 

## Figures and Tables

**Table 1:** Summary of participants, sampling, recruitment, and data collection.

Participant Group	Examples	Sampling and Recruitment Method	Data Collection
Women who have experienced a head injury (or diagnosed TBI) as a result of family violence	Women (aged 18+) who have experienced a head injury (or diagnosed TBI) as a direct result of family violence	Convenience sampling via key staff within community-based service providers and community groups. Purposive sampling, as required, to include underrepresented groups (such as young women)	Individual interview or small group discussion
Family members or caregivers of women	May include grandmother, parent, sister, guardian	Women who have experienced a head injury (or diagnosed TBI) as a direct result of family violence will be asked to nominate a family member or caregiver to participate in the project	Individual interview or small group discussion
Hospital staff	Aboriginal Liaison Officers, Aboriginal Leadership staff, specialists, and allied health staff	Purposive and snowball sampling	Individual interview or small group discussion
Community-based service providers	Family violence, legal and justice, health and community groups	Purposive and snowball sampling	Individual interview or small group discussion
Workshops	Community leaders, service providers, hospital staff, national advocacy group, and policymakers	A selection of service provider and hospital staff will be directly invited to take part in a one-day workshop	Workshop discussion

**Table 2:** Overview of the interview and discussion group topics.

Participant Group	Topics
Women who have experienced a head injury (or diagnosed TBI) as a direct result of family violence	Day to day living and challenges during the post-discharge period How women maintain roles involving taking care of family and cultural responsibilities How women re-construct their identity and life post-injury Motivation to engage with services and reconnect with life, community engagement, managing issues, and ability to build relationships with family, friends, and service networks Priorities, milestones, and outcomes that are important to women to achieve post-injury
Family members and carers	Day to day living during the post-discharge period for women, families and carers Challenges of caring for someone who has acquired a head injury connected to family violence Service and systems accessed by women, families, and carers Barriers and enablers for women, families, and carers to access services
Hospital staff	Experiences of women accessing hospital care and post-discharge processes Support for women in hospital and post-discharge including referral pathways Enablers and barriers for women to access hospital and health care Suggestions for improvements within services, systems, and policies
Community-based service providers	Service programs and the delivery of services Barriers and enablers for women to access services Challenges for service providers in terms of service delivery and sustainability Suggestion for reform and improvements for policy and service delivery

## Data Availability

Not applicable.
